# Cholelithiasis is an additional pre-pregnancy clinical risk factor for preeclampsia

**DOI:** 10.1007/s00404-023-06936-7

**Published:** 2023-01-28

**Authors:** Svitlana Arbuzova, Margaryta Nikolenko, David Wright, Howard Cuckle

**Affiliations:** 1 Eastern-Ukrainian Center for Medical Genetics and Prenatal Diagnosis, Mariupol & Kiev, Ukraine; 2 Institute of Health Research, University of Exeter, Exeter, England; 3 Faculty of Medicine, Tel Aviv University, Tel Aviv, Israel

**Keywords:** Screening, Prenatal, Pathogenesis, Marker

## Abstract

**Purpose:**

To investigate additional potential clinical risk factors for preeclampsia.

**Methods:**

This is a nested case–control study of preeclampsia and unaffected pregnancies. Cases were either from a prenatal screening database or from a national network of clinicians, and controls were from the same prenatal source. Preeclampsia was defined by international criteria which were endorsed by the Ukraine Ministry of Health. Questionnaires were used to record a range of pregnancy related factors, personal history of health conditions and family history, followed by a telephone interview to collect more details.

**Results:**

There were 103 cases, 56 from the prenatal database and 47 from the clinicians, and 480 controls from the database. The two types of case did not differ in terms of age, weight, BMI or parity. Known risk factors were more common in cases than controls. In addition there was a 17-fold higher prevalence of cholelithiasis in cases compared with controls (29.1% versus 1.7%), a highly statistically significant difference (*P* < 0.0001). There was also an 8.8-fold increase among the mothers of cases and controls (*P* < 0.0001), and if either the patient or her mother had the disease the increase was 6.4-fold (*P* < 0.0001). Including the father or sibling did not increase the relative risk.

**Conclusion:**

Cholelithiasis is a clinical risk factor for preeclampsia which has not previously been reported. If confirmed by additional studies it may have utility in routine prenatal screening and provide insight into the pathogenesis of preeclampsia.

## What does this study add to the clinical work

**Table:** 

Cholelithiasis is shown to be an additional clinical risk factors for preeclampsia. Incorporation into existing multi-marker screening protocols will improve performance and increase prevention.	

## Introduction

Preeclampsia (PE) is a leading cause of morbidity and mortality among mothers and infants worldwide. The serious life-threatening consequences of this disease have led to a large number of studies aimed at clarifying the pathogenesis of the condition, but it still remains uncertain.

In the last decade extensive research has been also devoted to preeclampsia screening as a means of reducing the prevalence of the disease through aspirin prophylaxis in those identified as high-risk. Substantial advances in screening have been made based on a multiparameter risk algorithm which uses selected risk factors together with biophysical and biochemical markers [1]. Previously, risk factors alone were used: strong factors such as a history of hypertensive disease in a previous pregnancy or chronic hypertension, chronic kidney disease, autoimmune disease and diabetes mellitus; moderate factors such as advanced maternal age, interpregnancy interval, body mass index and family history of preeclampsia [2]. However, combinations of all factors such as those recommended by National Institute for Health and Clinical Excellence [3] are both non-specific and insensitive. In contrast when selected obstetric factors (e.g., maternal age, parity, IVF conception, previous preeclampsia), clinical factors (e.g., chronic hypertension, diabetes, autoimmune disease) are combined with biophysical and biochemical marker levels a small high-risk group can be identified [4].

Although multi-marker screening for pre-term preeclampsia has been shown to be cost-effective it has not been adopted universally. In some localities there are insufficient resources available to initiate screening. The aim of the current study is to investigate potential additional clinical risk factors. If strong enough they would improve the cost-effectiveness of screening and may encourage wider adoption.

## Methods

### Patients

This is a retrospective nested case–control study. Cases comprised women who, in the period 2019–2021, had been diagnosed with preeclampsia according to the criteria established by ISSHP [5] and endorsed by the Ukraine Ministry of Health [6]. The criteria are: systolic blood pressure of 140 mm Hg or more and/or diastolic blood pressure of 90 mm Hg or more either pre-existing or developing after 20 weeks of gestation in a previously normotensive women, together with significant proteinuria (300 mg/24-h urine collection, or 30 mg proteins/mmol creatinine, or ≥ 2 + on dip-stick testing) and/or other signs of maternal endothelial dysfunction and/or uteroplacental dysfunction with intrauterine growth restriction.

There were two comparable sized series of cases. The first series (‘prenatal’) was derived from a database of women who had a first trimester Combined screening test for Down syndrome at the Eastern-Ukrainian Center for Medical Genetics and Prenatal Diagnosis. The second series (‘clinical’) was derived from a network of doctors in different regions of Ukraine. Having two series provided sufficient numbers of cases and was considered more representative of the country.

The prenatal database includes information collected at the time of screening and on follow-up of outcome for all pregnancies following delivery. The outcome information is specifically focused on aneuploidy and adverse outcomes of pregnancy. The network is associated with the Eastern-Ukrainian Center for Medical Genetics and Prenatal Diagnosis which provides expertise, training and conferences in prenatal diagnosis with doctors throughout Ukraine.

Controls comprised a consecutive series of women selected from the prenatal database at the Eastern-Ukrainian Center who gave their consent to the study. They had a negative Down syndrome screening test, did not have pregnancy complications and delivered a healthy child.

With the prenatal cases and the controls, consent to administer a questionnaire and carry out an interview was obtained routinely at the time of attending the Eastern-Ukrainian Center. With the clinical cases, individual consent was obtained locally following diagnosis.

A total of 103 cases, 56 prenatal 47 clinical, and 480 controls were included in the study. Among the preeclampsia cases, 78 (75.7%) were *diagnosed* preterm (before 37 weeks of gestation) and 62 (60.2%) were *delivered* preterm.

### Questionnaires and interview

The same structured questionnaire was used for cases and controls to record a range of pregnancy related factors, personal history of health conditions and history of the condition in her mother, father and siblings. The pregnancy related factors were: maternal age, weight, height, gravidity, parity, method of conception and outcome of previous pregnancies. The personal health conditions were: chronic hypertension, diabetes mellitus (non-pregnancy), thyroid disease, kidney disease, cardiovascular disease, systemic lupus erythematosus (SLE), anti-phospholipid syndrome (APS), infertility, ovarian or uterine fibroids and cysts, and polycystic ovarian syndrome (PCOS). History in the patient’s mother was also requested in relation to preeclampsia and infertility**.**

With the prenatal cases and the controls the questionnaire was routinely completed by the women when they first attended the Eastern-Ukrainian Center. At the same visit clinic staff checked for missing details and comprehensiveness. With the clinical cases, women were sent the questionnaire by e-mail, Viber or WhatsApp according to their individual preference.

All cases and controls were interviewed on the telephone by one of two authors (SA or MN). During the interview, items in the completed questionnaire were clarified, if needed, for personal conditions and those in her mother, father and siblings. At that time, the age of onset was requested and they were asked about conditions not included in the questionnaire. When an additional condition was specified, age of onset was requested. If, following the interview, further clarification was required the woman was contacted again by telephone.

### Statistics

Comparisons between the distributions of continuous variables were made using the Wilcoxon Rank Sum test. Differences in prevalence between cases and controls were assessed using a Chi-square Test; when comparisons were made between a small subgroup, Fisher’s Exact Test was used. All tests are two-sided and p-values < 0.05 were classified as statistically significant.

## Results

Table 1 compares the distribution of maternal age, weight, BMI (weight/height^2^) and parity in the current pregnancy for cases from the prenatal screening database and those identified by the network of clinicians. There were no statistically significant differences between the two types of case. The table also compares all cases combined with controls and parity was the only statistically significant variable (*P* < 0.0001); 35 cases (34.0%) and 345 controls (71.9%) had at least one previous pregnancy.

**Table 1:** Current pregnancy: maternal age, weight, BMI and parity in cases and controls

	Cases			Controls	*P*-value*	
	Prenatal (a) [*n* = 56]	Clinical (b) [*n* = 47]	All (c) [*n* = 103]	Prenatal (d) [*n* = 480]	(a) v. (b)	(c) v. (d)
Age (yrs)						
Mean	32.7	32.7	32.7	32.1	0.86	0.18
SD	4.79	5.69	5.19	5.31		
Median	34	32	33	32		
IQR	30–36	29–37	29–37	28–36		
Weight (kg)						
Mean	72.6	74.4	73.4	75.3	0.45	0.14
SD	16.6	14.7	15.7	9.77		
Median	70	72	71	74		
IQR	59–86	64–86	62–86	68–81		
BMI (kg/m^2^)						
Mean	26.6	27.3	26.9	27.0	0.39	0.59
SD	5.09	4.91	5.00	2.92		
Median	25.8	27.2	26.3	26.8		
IQR	22.2–30.1	24.1–30.5	23.0–30.1	25.2–28.9		
Parity						
Mean	0.411	0.489	0.447	0.919	0.61	< 0.0001
SD	0.654	0.718	0.682	0.710		
Median	0.00	0.00	0.00	1.00		
IQR	0.00–1.00	0.00–1.00	0.00–1.00	0.00–1.00		

*Wilcoxon Rank Sum Test (2-tail)

The proportion of cases conceived by IVF in the two case series and the controls were 7.1% (4/56) 10.6%, (5/47), and 1.0% (4/480), respectively; a statistically significant increase in all cases combined (*P* < 0.0001). Among women with previous pregnancies, one or more was affected by preeclampsia in 61.1% (11/18) and 23.5% (4/17), in the two cases series respectively (*P* < 0.05); there were none in the controls, a highly statistically significant difference compared to all cases combined (*P* < 0.0001).

In the two series of cases, the proportions diagnosed preterm were 69.6% (39/56) and 83.0% (39/47), respectively (*P* = 0.12); those delivered preterm were 66.0% (31/47) and 55.4% (31/56), respectively (*P* = 0.27).

Table 2 shows that the prevalence of pre-pregnancy clinical conditions in patients and/or their mothers were more common in cases than controls. Most are already known to be risk factors of preeclampsia. Chronic hypertension, diabetes, APS and SLE are currently incorporated into prenatal screening algorithms for pre-term preeclampsia [1]. In this study, diabetes was not a statistically significant risk factor but a history of the condition in the mothers of patients was significant. Thyroid, kidney and cardiovascular diseases, PCOS, hirsutism and ovarian or uterine fibroids and cysts are known clinical risk factors of preeclampsia although not included in screening algorithms. In this study, kidney disease was not a statistically significant risk factor but a history of the condition in mothers of patients was significant. There was no cardiovascular disease in patients, whilst a history of the condition in the mothers of patients was significantly more common in cases than controls.

**Table 2:** Pre-pregnancy clinical disorders reported in case and control patients, and their mothers

Clinical disorder	Patient			Patient’s mother		
	Cases [*n* = 103]	Controls [*n* = 480]	*P*-value	Cases [*n* = 103]	Controls [*n* = 480]	*P*-value
Used in prenatal screening						
Chronic hypertension	13 (12.6%)	6 (1.2%)	< 0.0001	43 (41.8%)	37 (7.7%)	< 0.0001
Diabetes^a^	2 (1.9%)	3 (0.6%)	0.22	17 (16.5%)	18 (3.8%)	< 0.0001
APS or SLE	4 (3.9%)	1 (0.2%)	< 0.005	0	0	−
Established risk factor but not used in prenatal screening						
Thyroid disease^b^	26 (25.2%)	38 (7.9)	< 0.0001	14 (13.6%)	20 (4.2%)	< 0.0002
Kidney disease^c^	11 (10.7%)	49 (10.2%)	0.89	14 (13.6%)	25 (5.2%)	< 0.002
Cardiovascular disease^d^	0	0	−	23 (22.3%)	15 (3.1%)	< 0.0001
Polycystic ovary syndrome	28 (27.2%)	19 (4.0%)	< 0.0001	9 (8.7%)	0	< 0.0001
Hirsutism	30 (29.1%)	17 (3.5%)	< 0.0001	9 (8.7)	3 (0.6)	< 0.0001
Ovarian/uterine fibroids/cysts	9 (8.7%)	14 (2.9%)	< 0.01	12 (11.6%)	28 (5.8%)	< 0.05
Potential new risk factor						
Gallstones	30 (29.1%)	8 (1.7%)	< 0.0001	47 (45.6%)	25 (5.2%)	< 0.0001

*APS* = anti-phospholipid syndrome, *SLE* systemic lupus erythematosus

^a^Among mothers with diabetes, two had type I; all other cases and controls had type II

^b^Among cases with thyroid disease, seven specifically reported hypothyroidism

^c^Among mothers with kidney disease, four cases and one control reported stones

^d^Among mothers with cardiovascular disease, 10 had heart attacks and strokes

Cholelithiasis (gallstones), a previously unreported clinical risk factor, was reported more frequently in cases. There was a 17-fold higher prevalence of this condition in cases compared with controls (29.1% versus 1.7%), a highly statistically significant difference (*P* < 0.0001). The relative risk of 17 had a 95% CI of 8.2–37. Gallstones were also more frequent in the mothers of cases. The table shows an 8.8-fold increase among the mothers of cases compared to mothers of controls (*P* < 0.0001) with 95% CI 5.7–14. Considering prenatal cases and controls only, the relative risks were 15 and 7.9 for patients and mothers (both *P* < 0.0001). There was no significant difference in the rate of gallstones in patients with pre-term or term preeclampsia: using gestation at onset 36.0% versus 26.9% (*P* = 0.38) or gestation at birth 31.7% versus 27.4% (*P* = 0.64).

The median age of cholelithiasis onset in cases was less than controls for the women themselves (25 versus 42 years, *P* < 0.0001) and their mothers (35 versus 42 years, *P* < 0.0001). Among the 16 cases where both the patient and her mother had a history of gallstones, in all except two the patient was diagnosed with the condition at an earlier age than the mother.

In the current pregnancy, information was available on diagnoses of intrahepatic cholestasis of pregnancy (ICP). There were 20 diagnoses among cases (19.4%) compared with 5 in controls (1.0%), a highly statistically significant difference (*P* < 0.0001). The median gestation of diagnosis was 35 weeks in cases and 36 weeks in controls. Among the cases with IPC, 11 (55%) had a history of gallstones and there were none in the controls (*P* < 0.05).

Table 3 shows the prevalence of gallstones in preeclampsia cases and unaffected controls according to the presence or absence of the other known clinical risks factors. There was an increased prevalence in cases in those with at least one risk factor or none. This was statistically significant when all clinical factors were used but when only the three clinical factors included in multi-marker screening were used it was only significant for the mothers of patients. Among the patients and controls with one or more of these factors there were only two with a history of gallstones, too few for statistical reliability.

**Table 3:** Prevalence of gallstones in women with and without other pre-pregnancy clinical risk factors for preeclampsia

	Patient			Patient’s mother		
	Cases	Controls	*P*-value	Cases	Controls	*P*-value
Risk factors used in prenatal screening						
One or more	2/18 (11.1%)	0/10 (0.0%)	0.27	8/18 (44.4%)	0/10 (0.0%)	< 0.02
None	28/85 (32.9%)	8/470 (1.7%)	< 0.0001	39/85 (45.9%)	25/470 (5.3%)	< 0.0001
All established risk factors						
One or more	22/67 (32.8%)	1/127 (0.8%)	< 0.0001	30/67 (44.8%)	8/127 (6.3%)	< 0.0001
None	8/36 (22.2%)	17/353 (2.0%)	< 0.0001	17/36 (47.2%)	17/353 (4.8%)	< 0.0001

Table 4 shows the relative risk of preeclampsia when history of gallstones is present in the patient, her mother and other close family. If either the patient or her mother had had gallstones, the relative risk of preeclampsia was 8.6 (*P* < 0.0001), lower than for the patient, but almost two-thirds of the cases were accounted for by such a history. Including the father or a sibling did not increase the relative risk.

**Table 4:** Gallstones in patient and/or first degree relatives among cases and controls

Gallstones	Cases [*n* = 103]	Controls [*n* = 480]	*P*-value
Patient and mother	16 (15.5%)	0	< 0.0001
Only patient	14 (13.6%)	8 (1.7%)	< 0.0001
Only mother	31 (30.1%)	25 (5.2%)	< 0.0001
Patient or mother	61 (59.2%)	33 (6.9%)	< 0.0001
Father	4 (3.9%)	3 (0.6%)	< 0.05
Sister	1 (1.0%)	1 (0.2%)	0.32
Brother	1 (1.0%)	0	0.18
Patient or father	32 (31.1%)	10 (0.2%)	< 0.0001
Patient or sister	31 (30.1%)	9 (1.9%)	< 0.0001
Patient or brother	31 (30.1%)	8 (1.7)	< 0.0001

## Discussion

There was a highly statistically significant association between cholelithiasis and preeclampsia. To our knowledge this is the first publication of such an association. A clinical history of gallstones could be included in algorithms used for the prediction of preeclampsia. An association was also found between cholelithiasis in the mothers of patients and preeclampsia. This too might be incorporated into screening algorithms.

The study recruited patients from throughout the Ukraine and not confined to a local population who might have different characteristics. However, since this was a hypothesis generating project the finding on cholelithiasis will need to be confirmed elsewhere.

The information on clinical conditions was obtained by both questionnaire and interview. This has the advantage of allowing double checking and refining the information provided. Although it was not possible to access medical records of women reporting having specific diseases the information regarding a personal history of gallstones is reasonably reliable. All interviewees were confident when reporting this diagnosis. The reliability of other clinical conditions among cases is less certain except for those in the ‘prenatal’ subgroup who, like the controls, underwent examination directly in the Eastern-Ukrainian Center and were more motivated to cooperate. Nonetheless, most of the known clinical risk factors for preeclampsia were found in the current study, confirming reliability in general. The information on clinical conditions in the mothers of cases and controls must be considered less reliable than when women reported their own conditions.

*P*-values < 0.05 were classified as statistically significant. When multiple comparisons are being made a more stringent cut-off might be adopted. This would not have influenced the finding on cholelithiasis since the level of significance was extreme (*P* < 0.0001) for both patients and their mothers.

The observation that a pre-pregnancy history of cholelithiasis is a risk factor for preeclampsia is consistent with impaired hepatobiliary function in affected pregnancies, evidenced by increased level of liver enzymes and dysregulated cholesterol homeostasis [7–9]. Moreover, chronic liver disease is an independent risk factor for preeclampsia [10] and this condition is predominantly associated with increased gallbladder wall thickness [11] which has been found in four studies of preeclampsia and eclampsia pregnancies; two case reports [12, 13] and two case series [14, 15]. The first report described two women presenting at with upper right quadrant pain, at 24 weeks gestation and postpartum, respectively; the gallbladder abnormality was found on ultrasound in each case [12]. In the second report, at day 4 postpartum following term delivery, the patient experienced right upper abdominal pain and a CT scan was performed which revealed enlarged heart, lung edema, bilateral pleural effusion, bilateral atelectasis and the gallbladder abnormality [13]. The first series comprised 32 pregnant women with acute right upper quadrant and epigastric pain at 24–36 weeks gestation; ultrasound examination at admission and postpartum found that 27 (84%) had gallbladder wall thickening [14]. The second series included 115 women who had an ultrasound of the biliary gallbladder, 93 in pregnancy and 22 immediately postpartum; 62 (54%) had gallbladder wall thickening [15].

Intrahepatic cholestasis is known to be common in preeclampsia pregnancies. A study of 78 women diagnosed with ICP, 54 singletons and 24 twins, compared the prevalence of preeclampsia with that in 200 singleton and 100 twin pregnancies. Rates were 7.4% and 1.5% respectively in singletons (*P* < 0.05); in twins they were 33% and 6.2% (*P* < 0.05) [16]. In another study of 180 women with ICP (162 singletons and 18 twins), the prevalence of preeclampsia was 7.8% compared with a reference group of 1618 pregnancies when it was 2.4% (*P* < 0.0001) [17]. A meta-analysis of five further studies derived a pooled odds ratio of 2.6 (95% CI 2.4–2.8) [18].

ICP is an indicator of liver and biliary diseases and in particular associated with gallstones; women with recurrent ICP have a high prevalence of gallbladder disease [19, 20]. Moreover, ICP induces placental oxidative stress, apoptosis, and vascular damage to the placenta that resembles that seen in preeclampsia [21–23]. Women with ICP are also at risk of other diseases associated with preeclampsia—cardiovascular disease, autoimmune conditions, diabetes mellitus [18, 24, 25], as well as poor obstetrical outcomes—preterm birth, stillbirth, intrauterine death associated with high levels of maternal serum bile acids [26, 27].

In the current study it is noteworthy that amongst those who had ICP, two had gallstones diagnosed in childhood, in their mothers aged in the 20 s, and the maternal grandmothers were diagnosed with hepatobiliar cancer. Although molecular genetic testing has not been performed, such clinical features and family history are pathognomonic for the low phospholipid-associated cholelithiasis syndrome (LPAC) [28]. Gallstones, LPAC and ICP have been linked to defects in the ATP-binding cassette (ABC) subfamily B transporter genes [29, 30]. This opens up a possible new direction for research into preeclampsia susceptibility genes.

The aim of this study was to investigate additional potential clinical risk factors for preeclampsia with a view to improving antenatal screening. The presence of gallstones in the woman being screened was found to be a strong risk factor, conferring a 17-fold relative risk and the association appears to be present in women with and without other clinical risk factors. A history of gallstones in the mother of the woman being screened also confers an increased risk, albeit less than that of the woman herself. Multi-marker screening algorithms could incorporate both risk factors taking account of the correlation between them as shown in Table 4. Currently, these algorithms do not take account of clinical risk factors, such as chronic hypertension, in family members. If doing so would improve predictive value this could be considered. Whilst obtaining reliable information about clinical conditions in relatives may be difficult, in the current study women seemed to be confident when reporting a history of gallstones in their mothers.

In conclusion, cholelithiasis is a clinical risk factor for preeclampsia which has not previously been reported. If confirmed by additional studies it may have utility in routine prenatal screening and provide insight into the pathogenesis of preeclampsia.

## Data Availability

The research data supporting this publication are available on request from the University of Exeter’s institutional repository at: 10.24378/exe.4485.
